# Persistent auditory hallucinations despite hearing aid use in bilateral sensorineural hearing loss without evidence of psychosis

**DOI:** 10.1177/2050313X261454846

**Published:** 2026-05-26

**Authors:** Bushra Elhusein, Nicole Willis, Sharfi Ahmed, Marco De Tubino Scanavino

**Affiliations:** 1 Department of Psychiatry, Schulich School of Medicine & Dentistry, Western University, London, ON, Canada; 2 London Health Sciences Centre, Victoria Hospital, ON, Canada; 3 Faculty of Medicine and Health Sciences, Omdurman Islamic University, Khartoum, Sudan; 4 Department of Epidemiology and Biostatistics, Schulich School of Medicine & Dentistry, Western University, London, ON, Canada; 5 Lawson Research Institute, St. Joseph’s Health Care London, London Health Sciences Centre Research Institute, ON, Canada

**Keywords:** case report, auditory hallucinations, sensorineural hearing loss, hearing aids, sensory deprivation

## Abstract

Auditory hallucinations are frequently linked to the onset of psychotic disorders, especially when accompanied by delusions or disorganized thinking. However, hallucinations may also arise from non-psychiatric causes. Hearing loss is often underrecognized as a contributing factor, since reduced auditory input can produce perceptual experiences that mimic psychosis. This case report presents a woman in her 50s with bilateral sensorineural hearing loss who experienced persistent auditory hallucinations for more than 3 years. She maintained insight, normal daily functioning, and exhibited no other features of a primary psychotic disorder. Neuroimaging was unremarkable, and multiple antipsychotic trials yielded minimal benefit. Despite improved auditory input with hearing aids, her hallucinations persisted, highlighting that symptoms related to sensory deprivation may not resolve immediately after hearing correction. This case illustrates how sensory deprivation-related hallucinations can be misdiagnosed as psychiatric illnesses, resulting in unnecessary interventions. It underscores the importance of early identification of sensory impairments and interdisciplinary collaboration when clinical presentations deviate from typical psychiatric conditions.

## Introduction

Auditory hallucinations (AHs) are seen as hallmark symptoms of primary psychotic disorders, especially schizophrenia and related conditions. However, AHs alone do not conclusively indicate psychosis. Studies show that hallucinations can appear in various non-psychotic medical, neurological, or sensory conditions.^1^ Hearing loss is a significant but often overlooked factor. Epidemiological studies report higher rates of AHs among individuals with hearing loss. Up to 16% of those with moderate impairment and almost 24% of those with severe impairment report hallucinatory experiences.^2^ These hallucinations can range from simple perceptions, such as faint murmurs or tones, to more complex phenomena, such as voices calling the patient’s name. Despite this known association, sensory deprivation hallucinations are often misunderstood.^3^ This may delay diagnosis and lead to inappropriate treatment.

Mechanisms behind hallucinations in hearing impairment are thought to be similar to those in other sensory deprivation conditions.^4^ Neuroimaging and electrophysiological studies show that reduced peripheral auditory input can lead to hyperexcitability in auditory cortical regions.^5^ Without normal sensory signals, intrinsic neural activity may be amplified or misread by higher brain centers. This can create perceptual experiences that seem like external sounds or voices. This matches the cortical disinhibition model used to explain Charles Bonnet syndrome in visual impairment. When deprived of expected sensory input, the perceptual system may create internal stimuli that feel external.^6^ These experiences can occur in otherwise healthy people and do not always signal psychiatric disorders.

Clinically, sensory-related hallucinations often differ from those in primary psychotic disorders. Patients typically have insight, recognizing these experiences as abnormal.^1^ They usually do not have delusions, disorganized thinking, negative symptoms, or cognitive decline. Functioning is mostly intact, and the clinical picture remains stable over time. However, AHs are frequently misread as signs of psychosis. This may result in misdiagnosis and unnecessary antipsychotic treatment.^7^ This overlap makes diagnosis harder, especially if hallucinations persist or cause distress, or if the patient’s hearing impairment is unrecognized. For many clinicians, hearing externally perceived voices prompts an automatic psychosis evaluation, often without considering sensory factors.

Most published case reports describe hallucinations resolving after treatment of hearing impairment. Treatment may include hearing aids, cochlear implants, or medical management of middle ear conditions.^8^ However, hallucinations may persist in some individuals despite sensory correction. This persistence challenges the assumption that sensory deprivation hallucinations resolve once auditory input is restored. In these cases, long-term cortical reorganization, heightened perception of internal noise, or altered attentional processes may lead to ongoing symptoms. Persistent hallucinations in this context are rarely reported, leading to a literature gap and uncertainty about optimal management strategies.^9^

This case adds to the limited evidence on persistent AHs in people with confirmed bilateral sensorineural hearing loss. The patient had preserved insight, stable function, normal neuroimaging, and no other psychotic symptoms. Thus, a primary psychotic disorder was deemed unlikely. Despite several antipsychotic trials, including risperidone, aripiprazole, and haloperidol, AHs did not change. Hearing aids greatly improved her hearing, but hallucinations persisted. Long-lasting symptoms, along with resistance to antipsychotic treatment and hearing correction, highlight the case’s clinical importance. It shows the need to consider sensory causes early in hallucination assessments. The case supports the value of interdisciplinary teamwork to prevent unnecessary or harmful interventions.

## Case presentation

A woman in her early 50s had no psychiatric or family history of mental illness. She reported 3 years of AHs that began gradually, with no clear trigger. At first, she heard her name called when she was alone at home in quiet settings. Later, she heard mumbling voices she could not understand. She consistently localized the voices as originating externally, describing them as sounds coming from outside her head rather than as internally generated thoughts. The voices did not speak to her, comment on her actions, or give orders. They were vague, brief, hard to interpret, and distressing. Background noise often reduced the intensity of the voices.

Over 2 years, the patient made several emergency visits and had short inpatient stays. Her symptoms were diagnosed as unspecified psychosis. These episodes led to multiple antipsychotic medication trials. Risperidone was initiated and titrated to 4 mg daily without improvement. Next, aripiprazole was given and increased to 20 mg daily, also without benefit. Then haloperidol was started and increased to 10 mg daily. Haloperidol did not alter the patient’s hallucinations. Nevertheless, the patient reported feeling “calmer” and less distressed, attributing this improvement to a stabilizing effect rather than any change in AHs. In light of this perceived benefit, haloperidol was continued, but the dose was reduced to 5 mg daily to minimize long-term extrapyramidal and other adverse effects while retaining the perceived calming benefit.

During admissions and outpatient visits, mental status exams showed preserved insight and logical thought. Speech was regular, affect was stable, and there were no delusions, paranoia, disorganization, or negative symptoms. The patient denied visual hallucinations, mood or cognitive changes, substance use, or agitation. She behaved normally and gave consistent symptom reports. Functioning stayed fully intact. She continued her full-time job, managed her household, maintained relationships, and had an active social life. There was no decline in self-care. Collateral information supported her account with no discrepancies.

The clinical history and behavioral assessments did not imply any intentional generation of symptoms or behavioral patterns typically associated with factitious disorders.

**Figure 1.**
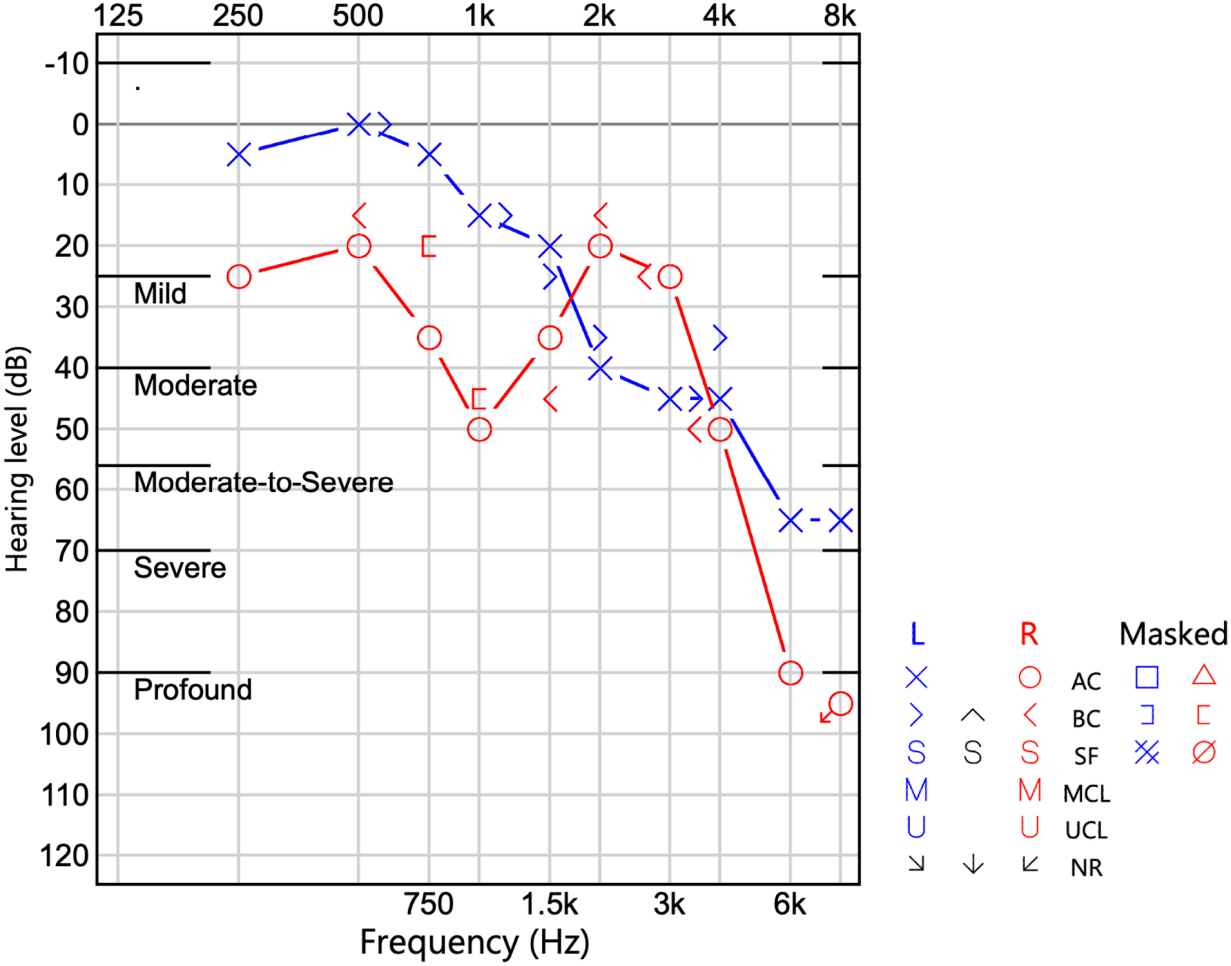
Pure-tone audiogram demonstrating bilateral sensorineural hearing loss.

During assessments, the clinicians noticed that she often leaned forward, cupped her ear, or asked for repetition. This led to an audiology referral ~4–6 months after her first contact with our team, and she was subsequently diagnosed with bilateral sensorineural hearing loss (Figure 1). The audiology report stated the following: “normal sloping to moderately-severe sensorineural hearing loss in the left ear, and mild sloping to profound sensorineural hearing loss with a 1000 Hz notch in the right ear.”

Approximately 2 months after audiological diagnosis, which corresponded to roughly 1 year after the initial psychiatric evaluation by our team, the patient was fitted with bilateral hearing aids. She reported consistent daily adherence to her hearing aids and was observed wearing them at every follow-up visit. Amplification produced a clear subjective improvement in environmental sound perception and conversational clarity. Formal aided audiometric reassessment and dedicated speech-in-noise or speech-discrimination testing were not performed during follow-up; nevertheless, her functional gain from amplification was clinically evident, and the otolaryngologist’s expected aided benefit profile was consistent with her reported experience. Despite this, the hallucinations remained unchanged in frequency, phenomenology, and associated distress.

Given the persistence of hallucinations despite multiple antipsychotic trials and the presence of significant sensorineural hearing loss, the clinical formulation was reconsidered. The patient demonstrated preserved insight, intact functioning, normal neuroimaging, and no additional psychotic features such as delusions, disorganization, or negative symptoms. In the context of documented hearing impairment, these findings supported the interpretation that the AHs were most consistent with sensory deprivation related to hearing loss rather than a primary psychotic disorder. At follow-up over the subsequent year, the hallucinations persisted without progression. The patient’s functioning, insight, and mental health remained stable.

**Figure 2.**
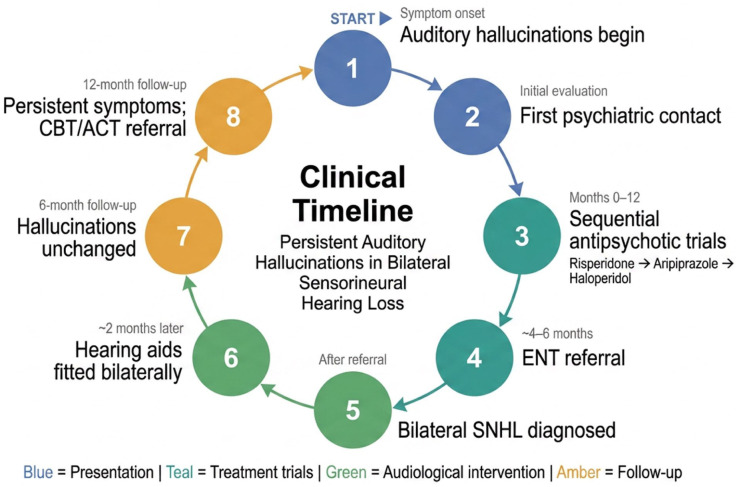
Chronological clinical timeline of the patient’s presentation, diagnostic workup, and follow-up. Color-coded phases highlight the progression from repeated psychiatric admissions and unsuccessful antipsychotic trials to audiological diagnosis, bilateral hearing-aid fitting, and persistence of auditory hallucinations across 12 months of follow-up.

A chronological summary of the clinical course is presented in Figure 2.

## Investigations

Audiology assessment identified bilateral sensorineural hearing loss. Tympanometric results were normal. Hearing aids were prescribed, leading to improved auditory acuity.

Neuroimaging, which included computed tomography of the head and magnetic resonance imaging of the brain, showed normal results. Laboratory evaluations, including a complete blood count, metabolic profile, thyroid function tests, and levels of vitamin B12 and folate, as well as inflammatory markers, were all within normal limits.

Clinical examination revealed no neurological deficits. The patient had previously been reviewed by a neurologist after the onset of hallucinations and was neurologically cleared. There was no history of seizures, episodic focal neurological symptoms, or features suggestive of ictal phenomena; an electroencephalogram was therefore deemed not clinically indicated by the neurology team.

Formal structured cognitive screening was not performed as there were no clinical indicators of cognitive impairment. The patient remained alert and fully oriented to time, place, and person across all encounters, with attention, short- and long-term memory, language, and executive functioning intact on clinical interview.

## Differential diagnosis

The differential diagnosis for isolated AHs included primary psychotic disorders such as schizophrenia; however, this was deemed unlikely due to the patient’s preserved insight, absence of delusions, disorganization, or negative symptoms, stable functioning over 3 years, and lack of progression despite ineffective antipsychotic treatment. Mood disorders with psychotic features were excluded, given the absence of depressive or manic episodes.

Psychosis secondary to neurological or other medical conditions was excluded based on normal neuroimaging, unremarkable laboratory findings, and absence of neurological signs. Substance-induced psychosis was ruled out due to a negative substance use history.

A broader range of alternative contributors was systematically considered. Sleep-related hallucinatory phenomena, including hypnagogic and hypnopompic hallucinations, were explored on direct questioning; the voices occurred throughout wakeful hours and bore no temporal relationship to sleep–wake transitions. A detailed history identified no significant psychological trauma, dissociative symptoms, or post-traumatic features. The patient denied tinnitus as well as any musical, melodic, or rhythmic quality to the hallucinations, making musical hallucinosis unlikely. Temporal-lobe epilepsy and other ictal causes were considered unlikely given the absence of seizure history, normal neurological examination, and prior neurology review.

Considering the confirmed moderate to severe bilateral sensorineural hearing loss, preserved insight, non-thematic and straightforward hallucination phenomenology, external localization of the voices, and stable functioning, the most consistent diagnosis was AHs related to sensory deprivation secondary to hearing impairment.

## Outcome and follow-up

At 6- and 12-month follow-ups, the hallucinations persisted without progression. The patient remained functionally independent and employed. No additional psychotic or mood symptoms were observed. The calming effects of the reduced haloperidol dose persisted, allowing it to be further decreased to 2 mg daily. This adjustment aimed to minimize unnecessary side effects while still maintaining the same calming effects. Despite improved hearing, the hallucinations remained resistant to auditory correction. The patient expressed frustration but reported no safety concerns related to treatment.

Given her prolonged symptomatology and limited response to pharmacotherapy, psychotherapy was discussed as an adjunctive strategy. Psychoeducation was provided on the link between sensory impairment and perceptual phenomena, emphasizing that the therapeutic goal would be to reduce distress and improve coping rather than to eliminate the hallucinations. To the best of our knowledge, following a comprehensive review of the existing literature, no studies have been identified that specifically investigate the efficacy of cognitive behavioral therapy (CBT) or acceptance and commitment therapy (ACT) for persistent AHs in individuals with hearing impairment who do not respond to antipsychotic treatment. However, in individuals with psychosis experiencing persistent AHs unresponsive to antipsychotics, CBT has demonstrated moderate and consistent evidence in reducing hallucination-related distress. A meta-analysis of formulation-based CBT reported a pooled effect size of ~0.44–0.49 for AHs with low heterogeneity.^10^ Thomas et al. highlighted that contemporary CBT and ACT approaches explicitly target beliefs about voices’ perceived power, threat, controllability, and identity and teach coping and acceptance strategies, with sustained benefit even when the phenomenology persists.^11^ The patient agreed to referral for CBT incorporating elements of ACT, focusing on psychoeducation, symptom appraisal reframing, and adaptive coping. She remains on the therapy waiting list at the time of submitting this case.

## Discussion

AHs commonly occur in primary psychotic disorders, particularly schizophrenia. However, many patients experience hallucinations without underlying psychotic illness, often in association with sensory impairments.^2^ This case underscores the diagnostic challenge posed by isolated hallucinations occurring without delusions, disorganization, or functional decline. The patient’s preserved insight, normal neuroimaging, stable functioning, and lack of progression support a nonpsychiatric cause. Persistent and distressing hallucinations resulted in repeated hospital admissions and antipsychotic trials before recognition of the hearing impairment’s significance, a scenario not frequently encountered in clinical practice.

**Figure 3.**
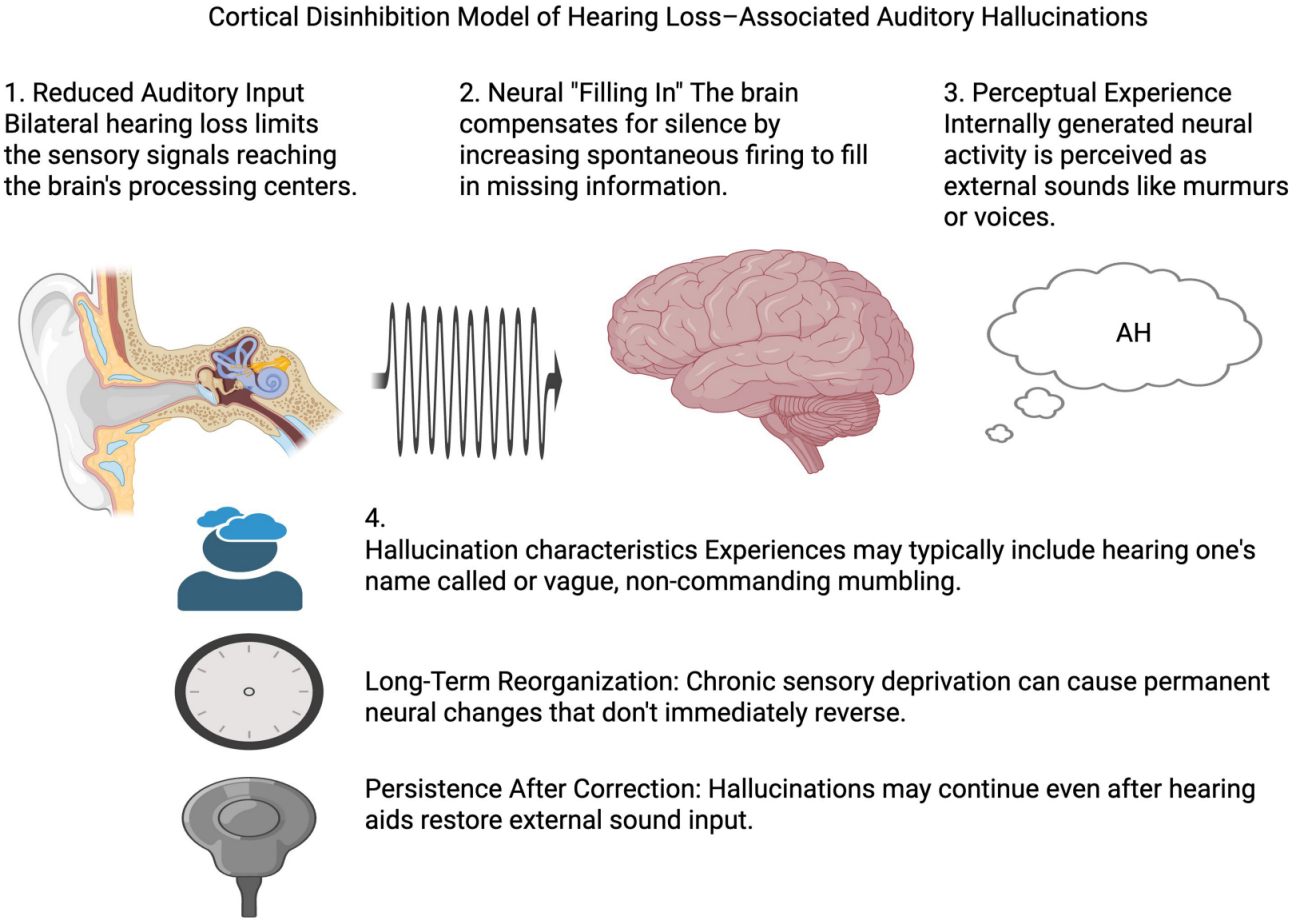
Conceptual illustration of the cortical disinhibition model of hearing loss-associated auditory hallucinations, adapted for teaching purposes. The figure is illustrative and does not represent case-specific data.^12^

Hallucinations associated with hearing loss have been documented for over a century, and neuroscience research has provided plausible mechanisms. Reduced auditory input can induce hyperexcitability in auditory cortical regions, analogous to phenomena such as central sensitization in phantom limb pain, tinnitus, and Charles Bonnet syndrome.^4,6^ The brain compensates for decreased sensory input by increasing spontaneous neural firing or “filling in” missing information, resulting in perceptions of voices, murmurs, or other sounds.^2^ Patients typically report simple, non-threatening auditory experiences, such as hearing their name called or faint voices, consistent with the present case. This mechanism is illustrated conceptually in Figure 3, which is provided as a teaching schematic rather than as case-specific evidence (Figure 3).^12^

The prevalence of AHs in hearing-impaired populations has been characterized across several cohort studies of varying size and design. In a large cross-sectional study of more than 1000 hearing-impaired adults, Linszen et al. found that 16.2% reported AHs in the preceding 4 weeks, with prevalence rising to ~24% among those with the most severe impairment.^2^ Cole et al., in a clinic-based study of elderly audiology patients, reported an even higher prevalence of 32.8% and documented a phenomenological spectrum ranging from elementary percepts such as buzzing and ringing to complex verbal hallucinations including recognizable voices and conversational speech.^13^ Despite this evidence base, clinical awareness remains limited. Marschall et al. surveyed psychiatrists and audiologists and found that knowledge of hallucinations in hearing impairment was low across both specialties, contributing to diagnostic delay and frequent misattribution of symptoms to primary psychotic disorders.^3^ With respect to persistence following auditory rehabilitation, the available data suggest that correction of the sensory deficit does not uniformly resolve hallucinatory phenomena. Brüggemann et al. reported that 33% of cochlear implant recipients experienced musical ear syndrome, with a substantial proportion reporting symptoms both before and after implantation.^14^ Similarly, Millen et al. recently described two patients with bilateral acquired hearing loss and no prior psychiatric history whose AHs were sufficiently complex to trigger multiple antipsychotic trials before the hearing loss was recognized.^15^ More recent case-level evidence further documents sustained improvement, or worsening hallucinations despite timely hearing-aid fitting and adherent use, suggesting that peripheral amplification alone is insufficient to resolve the perceptual disturbance in a subset of individuals.^8,16,17^

A significant challenge arises when hallucinations persist despite effective auditory rehabilitation. Although many patients improve following restoration of auditory input with hearing aids or cochlear implants, this is not universal. Neuroplastic changes resulting from prolonged sensory deprivation may persist after partial hearing restoration. Neuroimaging studies demonstrate that the auditory cortex can undergo long-term reorganization in chronic hearing loss, and these alterations do not always reverse with improved peripheral hearing.^18^ Consequently, spontaneous auditory perceptions may continue despite correction of the sensory deficit. This phenomenon explains the persistence of hallucinations despite objective hearing improvements. Ongoing hallucinations after sensory correction do not necessarily indicate psychosis; rather, they represent a recognized consequence of prolonged auditory deprivation.^18^

Distinguishing these experiences from primary psychotic disorders is essential. In psychosis, hallucinations commonly co-occur with delusions, disorganized thinking, negative symptoms, and functional decline. Over time, the clinical presentation evolves, and insight into symptoms may diminish.^19^ None of these features was present in this patient. Her capacity to reflect on symptoms, seek assistance, maintain employment, and manage responsibilities supports a nonpsychiatric etiology. The lack of response to antipsychotic treatment further corroborates this distinction. Antipsychotic medications primarily target dopaminergic pathways central to schizophrenia^20^, but are largely irrelevant to sensory deprivation hallucinations. The patient’s report of feeling calmer on haloperidol likely reflects nonspecific anxiolytic effects rather than a direct effect on hallucinations.

The patient’s consistent experience of voices as externally localized, together with their simple non-commanding content, preserved reality testing, and absence of delusional elaboration, is phenomenologically more characteristic of sensory-deprivation-related auditory phenomena than of auditory verbal hallucinations typically described in primary psychotic disorders.^21^

This highlights the importance of a nuanced management approach. When hallucinations result from sensory deprivation, eliminating them may not be possible.^9^ Treatment focuses on reducing distress, strengthening coping mechanisms, and enhancing quality of life. The patient’s acceptance of psychotherapy aligns with evidence supporting cognitive-behavioral and acceptance-based approaches, which help patients understand symptoms, reframe interpretations, and lessen emotional burden, even if perceptual experiences persist.^11^

This case illustrates several key lessons. Early audiological assessment should be included when evaluating isolated AHs, especially when phenomenology aligns with sensory deprivation experiences. Misdiagnosis can lead to unnecessary antipsychotic exposure, and repeated medication trials may hinder the identification of the actual cause. Input from psychiatry, audiology, and neurology is essential to avoid diagnostic delays. Persistent hallucinations after correcting hearing impairment should not indicate psychosis. Instead, they may suggest residual cortical hyperexcitability or enduring perceptual patterns that slowly resolve.^9^

The persistence of hallucinations after improvement in auditory acuity does not contradict a sensory-related mechanism. Population studies show that auditory verbal hallucinations can occur in healthy individuals, often with stable phenomenology without progression to psychotic illness. de Leede-Smith and Barkus reported that many people experience intermittent or persistent hallucination-like phenomena throughout their lives, independent of psychiatric conditions.^22^ Their review showed that hallucinations are not inherently pathological and may arise from various cognitive, perceptual, or sensory mechanisms. The continuation of hallucinations can be understood as part of a nonpsychotic hallucinatory spectrum, initially triggered by auditory deprivation but sustained through long-lasting perceptual patterns. This supports that the lack of improvement with hearing aids does not necessarily indicate a primary psychotic disorder (Supplemental Material).

## Limitations and future directions

Several limitations should be acknowledged. As a single-case report, the findings are descriptive, hypothesis-generating, and not generalizable to the wider population of patients with sensorineural hearing loss and AHs. Formal aided audiometry and objective speech-in-noise or speech-discrimination testing were not performed after hearing aid fitting, which limits quantification of the functional gains from amplification. Objective measures of cortical auditory processing (such as auditory-evoked potentials or functional neuroimaging) could have offered mechanistic insight into residual cortical hyperexcitability. Priority areas for future investigation include prospective longitudinal studies of hallucination persistence following auditory rehabilitation, identification of clinical or neurophysiological predictors of non-response to amplification, and controlled trials evaluating cognitive-behavioral and acceptance-based psychotherapies in sensory-deprivation-related hallucinations.

This case contributes to the literature on persistent AHs despite hearing rehabilitation. It emphasizes the importance of considering sensory etiologies when diagnosing hallucinations and adopting individualized approaches that prioritize patient safety, insight, and functioning. The patient’s stable course, preserved insight, and intact functioning distinguish her presentation from primary psychotic disorders. This case demonstrates that hallucinations are not synonymous with psychosis and that interdisciplinary assessment can prevent prolonged unnecessary treatment.

## Learning points

- AHs are not invariably indicative of psychosis; sensory impairment represents a significant non-psychiatric etiology.
- Persistent hallucinations may continue even after correction of hearing loss due to residual cortical reorganization.
- Lack of response to antipsychotic treatment should prompt diagnostic re-evaluation before labeling the condition as treatment-resistant.
- Early audiological assessment may help prevent unnecessary antipsychotic exposure and misdiagnosis.
- External localization of voices, preserved insight, and stable functioning should alert clinicians to non-psychotic hallucinatory phenomena and a possible sensory etiology.

## Supplemental Material

sj-docx-1-sco-10.1177_2050313X261454846 – Supplemental material for Persistent auditory hallucinations despite hearing aid use in bilateral sensorineural hearing loss without evidence of psychosis Supplemental material, sj-docx-1-sco-10.1177_2050313X261454846 for Persistent auditory hallucinations despite hearing aid use in bilateral sensorineural hearing loss without evidence of psychosis by Bushra Elhusein, Nicole Willis, Sharfi Ahmed and Marco De Tubino Scanavino in SAGE Open Medical Case Reports
