# Supplementary material for: Persistent auditory hallucinations despite hearing aid use in bilateral sensorineural hearing loss without evidence of psychosis
Source: SAGE Open Med Case Rep. 2026 May 26;14:2050313X261454846. doi: 10.1177/2050313X261454846 (PMC13213105; doi:10.1177/2050313X261454846)
Supplement: sj-docx-1-sco-10.1177_2050313X261454846 – Supplemental material for Persistent auditory hallucinations despite hearing aid use in bilateral sensorineural hearing loss without evidence of psychosis [file sj-docx-1-sco-10.1177_2050313X261454846.docx]

| **Topic** | **Item** | **Checklist item description** | **Author Confirmation** |  |
| --- | --- | --- | --- | --- |
| **Title** | **1** | The words "case report" should be in the title along with the area of focus | Yes — article type: Case Report.  Title: "Persistent Auditory Hallucinations Despite Hearing Aid Use in Bilateral Sensorineural Hearing Loss Without Evidence of Psychosis."  Area of focus (auditory hallucinations in sensorineural hearing loss) is clearly stated. The descriptor "case report" is provided in the article-type designation per journal convention. |  |
| **Key Words** | **2** | Four to seven key words—including "case report" as a key word | Yes. Proposed key words to be listed below the abstract:  case report; auditory hallucinations; sensorineural hearing loss; hearing aids; sensory deprivation. |  |
| **Abstract** | **3a** | Background: What does this case report add to the medical literature | Yes — Abstract, opening sentences.  The case illustrates that persistent auditory hallucinations in bilateral sensorineural hearing loss can mimic primary psychosis and may not resolve following hearing-aid fitting, highlighting a recognised but under-reported clinical pattern. |  |
|  | **3b** | Case summary (1 paragraph): chief complaint, diagnoses, interventions, and outcomes | Yes — Abstract.  Woman in her fifties with 3+ years of persistent auditory hallucinations; bilateral sensorineural hearing loss identified on audiometry; multiple antipsychotic trials ineffective; bilateral hearing aids produced subjective auditory benefit but hallucinations persisted; preserved insight and functioning throughout. |  |
|  | **3c** | Conclusion: What are the main "take-away" lessons from this case? | Yes — Abstract, closing sentences, and the Learning Points section.  Sensory-deprivation–related auditory hallucinations can be misdiagnosed as psychiatric illness, leading to unnecessary antipsychotic exposure; early audiological assessment and interdisciplinary collaboration are essential. |  |
| **Introduction** | **4** | How does this case inform healthcare delivery—with references (1–2 paragraphs) | Yes — Introduction section (four paragraphs).  Frames the case within the prevalence and phenomenology of hallucinations in hearing impairment (refs 1, 2, 3), the cortical disinhibition / deafferentation mechanism shared with Charles Bonnet syndrome (refs 4, 5, 6), the clinical challenges in differentiating sensory-related from primary psychotic hallucinations (ref 7), and the limited literature on persistence after sensory correction (refs 8, 9). |  |
| **Timeline** | **5** | Relevant information from this case report organized into a timeline (table or figure) | Yes — Figure 3 (newly added in revision).  Chronological clinical timeline summarising symptom onset, initial psychiatric evaluation, sequential antipsychotic trials (risperidone, aripiprazole, haloperidol), audiology referral, audiogram findings, bilateral hearing-aid fitting, and 6- and 12-month follow-ups, colour-coded by phase (pre-diagnosis, recognition, intervention, follow-up). Cross-referenced in the Case Presentation. |  |
| **Patient Information** | **6a** | De-identified demographic and other patient specific information | Yes — Case Presentation, opening sentence.  "A woman in her early 50s had no psychiatric or family history of mental illness." No identifying details included; consent for publication of anonymized information obtained. |  |
|  | **6b** | Chief complaint (what prompted this patient visit) | Yes — Case Presentation.  Three-year history of persistent auditory hallucinations; initial presentation with voices calling her name in quiet settings, progressing to mumbling voices; consistently localised as external. |  |
|  | **6c** | Relevant medical and psychosocial history (including interventions and outcomes) | Yes — Case Presentation.  No prior psychiatric or family history; preserved employment, household management, relationships, and social activity throughout; multiple ER visits and brief inpatient stays with sequential antipsychotic trials; intact insight and reality testing across encounters. |  |
| **Physical Exam** | **7** | Relevant physical examination findings | Yes — Case Presentation and Investigations.  Mental status examinations: preserved insight, logical thought, normal speech, stable affect, no delusions/paranoia/disorganisation/negative symptoms.  Clinical observation of hearing difficulty (leaning forward, cupping ear, requesting repetition) that prompted audiology referral.  Neurological examination: no deficits; prior neurology clearance. Cognitive examination: alert, fully oriented, intact attention/memory/language/executive function. |  |
| **Diagnostic Assessment** | **8a** | Diagnostic evaluations (such as laboratory testing, imaging, surveys) | Yes — Investigations section.  Audiology with pure-tone audiometry (Figure 1) and tympanometry; CT head and MRI brain (normal); CBC, metabolic profile, TSH, vitamin B12, folate, inflammatory markers (all within normal limits); prior neurology review. |  |
|  | **8b** | Diagnoses (consider tables/figures linking assessment with diagnoses and interventions) | Yes — Investigations, Differential Diagnosis, and Figure 2.  Primary diagnosis: auditory hallucinations related to sensory deprivation secondary to bilateral sensorineural hearing loss. Figure 2 illustrates the cortical disinhibition model; Figure 1 documents the audiogram; Figure 3 integrates assessment, diagnosis, and intervention chronologically. |  |
|  | **8c** | Diagnostic reasoning including other diagnoses considered and diagnostic challenges | Yes — Differential Diagnosis section (systematically expanded in revision).  Alternatives evaluated: primary psychotic disorders (schizophrenia), mood disorder with psychotic features, medical/neurological psychosis, substance-induced psychosis, temporal-lobe epilepsy and ictal phenomena, hypnagogic/hypnopompic hallucinations, trauma/dissociative disorders, tinnitus and musical hallucinosis, cognitive decline, factitious disorder, and malingering. Diagnostic reasoning and challenges (initial misclassification, three ineffective antipsychotic trials) are transparently documented. |  |
|  | **8d** | Prognostic characteristics (such as staging in oncology) where applicable | Not applicable to this non-oncological, non-staged presentation.  Prognostic context is instead addressed narratively: stability of symptoms over 12 months of adherent amplification, preserved insight and functioning, and the expectation per the literature (refs 18, 22) that residual cortical reorganisation may sustain hallucinations despite peripheral sensory restoration. |  |
| **Interventions** | **9a** | Types of intervention (such as pharmacologic, surgical, preventive, self-care) | Yes — Case Presentation and Outcome and Follow-Up.  Pharmacologic: sequential antipsychotic trials (risperidone, aripiprazole, haloperidol).  Device-based: bilateral hearing aids.  Psychological (planned): CBT incorporating elements of ACT; psychoeducation. |  |
|  | **9b** | Intervention administration (such as dosage, strength, duration) | Yes — Case Presentation.  Risperidone titrated to 4 mg daily (no benefit); aripiprazole titrated to 20 mg daily (no benefit); haloperidol titrated to 10 mg daily, subsequently reduced to 5 mg daily maintenance for its calming effect.  Bilateral hearing aids fitted ~2 months after audiological diagnosis (≈1 year after initial psychiatric evaluation); daily adherence observed at every follow-up. |  |
|  | **9c** | Changes in intervention (with rationale) | Yes — Case Presentation and Outcome and Follow-Up.  Sequential antipsychotic switches driven by lack of symptomatic response. Haloperidol dose reduced from 10 to 5 mg daily to minimise long-term extrapyramidal risk while maintaining nonspecific anxiolytic benefit.  Reformulation from "unspecified psychosis" to "sensory-deprivation-related auditory hallucinations" once bilateral sensorineural hearing loss was confirmed.  Treatment plan expanded to include CBT/ACT referral due to persistent symptoms despite amplification. |  |
|  | **9d** | Other concurrent interventions | None beyond those listed.  No psychotropic polypharmacy, no somatic treatments (ECT/rTMS), no substance use. |  |
| **Follow-up and Outcomes** | **10a** | Clinician and patient-assessed outcomes (when appropriate) | Yes — Outcome and Follow-Up.  Clinician-assessed: hallucinations persisted unchanged in frequency, phenomenology, and associated distress at 6 and 12 months; insight, mental state, and functioning remained stable.  Patient-assessed: subjective improvement in environmental sound perception and conversational clarity with hearing aids; frustration regarding persistence of voices; calming effect on haloperidol 5 and 2 mg; no safety concerns. |  |
|  | **10b** | Important follow-up diagnostic evaluations | Addressed — Case Presentation and Limitations and Future Directions.  Formal aided audiometric reassessment, dedicated speech-in-noise and speech-discrimination testing, and objective measures of cortical auditory processing (auditory-evoked potentials, functional neuroimaging) were not performed and are explicitly acknowledged as a limitation. |  |
|  | **10c** | Assessment of intervention adherence and tolerability | Yes — Case Presentation and Outcome and Follow-Up.  Hearing aids: self-reported daily use, corroborated by observation at every visit.  Haloperidol 5 mg daily: tolerated without adverse events; subjective "calmer" effect reported. |  |
|  | **10d** | Adverse and unanticipated events | Yes — Case Presentation and Outcome and Follow-Up.  No adverse medication reactions reported. Unanticipated finding: persistence of hallucinations despite adherent bilateral amplification — a recognised but under-reported clinical course that motivated the reformulation and psychotherapy referral. |  |
| **Discussion** | **11a** | Strengths and limitations in your approach to this case—with references | Yes — Limitations and Future Directions paragraph (newly added in revision) and Discussion.  Strengths: prospective longitudinal follow-up; comprehensive exclusion of alternative diagnoses; multidisciplinary input (psychiatry, audiology, neurology); phenomenological rigour (refs 2, 3, 13, 21).  Limitations: single-case, hypothesis-generating, non-generalizable design; absence of formal aided audiometry, speech-in-noise testing, auditory-evoked potentials, and structured cognitive screening (refs 18, 22). |  |
|  | **11b** | Conclusions and rationale (including possible causes for outcomes) | Yes — Discussion (final paragraphs) and Learning Points.  Outcome interpretation: persistence of hallucinations is attributed to residual cortical reorganisation and enduring perceptual patterns following prolonged auditory deprivation (refs 6, 18), consistent with the nonpsychotic hallucinatory spectrum described by de Leede-Smith and Barkus (ref 22).  The clinical conclusion supports early audiological assessment, cautious use of antipsychotics in atypical hallucinations, and integration of CBT/ACT-based psychotherapy for distress reduction (refs 10, 11). |  |
| **Patient Perspective** | **12** | When appropriate include the patient's perspective on this episode of care | Yes — Outcome and Follow-Up.  Patient expressed frustration at the persistence of hallucinations despite hearing-aid use but reported no safety concerns. She reported subjective benefit from amplification (improved conversational clarity) and a calming effect from haloperidol, distinct from any change in the voices themselves. She agreed to CBT/ACT referral and remains on the waiting list at the time of submission. |  |
| **Informed Consent** | **13** | Patient informed consent is likely to be required by a journal (or your institution) prior to publication | Yes — Consent to Participate and Consent for Publication statements.  Written informed consent was obtained from the patient for inclusion in this report and for publication of her anonymized clinical information. |  |
| **Other** | **14** | IRB approval as indicated or needed; Acknowledgement section; Competing Interests; Funding | Yes — Ethical Considerations, Declaration of Conflicting Interests, and Funding statements.  Ethical approval: institutional policy does not require ethical approval for individual case reports; this is stated explicitly.  Competing interests: none declared.  Funding: no financial support received.  Acknowledgements: none. ORCID iDs provided for B. Elhusein and M. De Tubino Scanavino. |  |

**CARE Checklist**

Manuscript ID: SOMCR-26-0186

*Title: Persistent Auditory Hallucinations Despite Hearing Aid Use in Bilateral Sensorineural Hearing Loss Without Evidence of Psychosis*

Journal: SAGE Open Medical Case Reports
